# Anatomical evaluation and cone-beam computed tomography of some maxillary nerve block approaches in dog

**DOI:** 10.1016/j.vas.2026.100599

**Published:** 2026-02-14

**Authors:** Kaveh Khazaeel, Anahita Memardezfouli, Hadi Imani Rastabi, Seyed Arman Mohagheghi

**Affiliations:** a Department of Basic Sciences, Division of Anatomy and Embryology, Faculty of Veterinary Medicine, Shahid Chamran University of Ahvaz, Ahvaz, Iran; b Stem Cells and Transgenic Technology Research Center (STTRC), Shahid Chamran University of Ahvaz, Ahvaz, Iran; c Department of Veterinary Clinical Sciences, Faculty of Veterinary Medicine, Shahid Chamran University of Ahvaz, Ahvaz, Iran; d Department of Oral and Maxillofacial Radiology, School of Dentistry, Ahvaz Jundishapur University of Medical Science, Ahvaz, Iran

**Keywords:** Maxillary nerve, Local anesthesia, Anatomical comparison, Cone-beam computed tomography, Dog

## Abstract

Effective pain management is a cornerstone of modern veterinary surgical care, with regional nerve blocks as a crucial tool for targeted analgesia. The maxillary nerve, a major sensory branch of the trigeminal nerve, innervates numerous facial and oral structures, making its blockade essential in oral and maxillofacial procedures in dogs. Despite several described approaches namely the subzygomatic, infraorbital, and maxillary tuberosity techniques, there remains limited comparative anatomical and radiological data to guide clinical decision-making. This study evaluated these three approaches using anatomical dissection and cone-beam computed tomography (CBCT) in six canine cadavers (*n* = 6). Each received an injectate (methylene blue, iohexol, and bupivacaine) via the three techniques. Pre- and post-injection CBCT scans were analyzed using NNT Viewer v3 and OnDemand3D software, measuring contrast spread in sagittal and dorsal planes (1- and 10-mm slices) and correlating with dissections. Results demonstrated that the infraorbital approach had significantly higher staining of the maxillary nerve compared to the maxillary tuberosity and subzygomatic approaches (*P* < 0.05). The maximum amount of diffusion of the contrast medium along the maxillary nerve was for the infraorbital approach. Sagittal (10 mm) and dorsal (1 mm) sections had the highest compliance with the length of dye diffusion measured in the anatomical sections. In the maxillary tuberosity approach, sagittal and dorsal views with a thickness of 10 mm had the highest compliance with the length of dye diffusion measured in the anatomical sections. The infraorbital method provided the most consistent and effective blockade, suggesting its preferential use in clinical practice for optimized analgesia.

## Highlights

• Infraorbital approach showed greatest maxillary nerve staining and diffusion depth.
• CBCT accurately demonstrated the spread of the injection and correlated with the dissection.
• Infraorbital method provides most consistent and effective maxillary blockade.
• Anatomical dissection confirmed the imaging and injection findings.

## Introduction

Effective pain management is a fundamental component of veterinary clinical care, especially in surgical and dental procedures (Epstein et al., 2015). The use of local anesthetic techniques, including regional nerve blocks, has gained widespread acceptance due to their ability to provide targeted, cost-effective analgesia with minimal equipment requirements (Grubb & Lobprise, 2020). Administering anesthetics in close proximity to peripheral nerves ensures adequate nerve blockade, significantly reducing perioperative and postoperative pain Viscasillas et al. (2013). Among companion animals, particularly dogs and cats, regional blocks have become routine during oral and maxillofacial interventions (Pascoe & Chohan, 2020). Techniques such as inferior alveolar, infraorbital, and maxillary nerve blocks are commonly employed to facilitate dental extractions, maxillary surgeries, and related procedures (Viscasillas et al., 2013). The maxillary nerve, a sensory branch of the trigeminal nerve, innervates the maxillary dentition, surrounding soft and hard tissues, the nasal cavity, and part of the orbit (Veres‐Nyéki, 2022). Anatomically, it exits the skull via the orbital fissure, travels along the dorsal surface of the medial pterygoid muscle toward the pterygopalatine fossa, and continues through the maxillary foramen into the infraorbital canal (Gioso et al., 2005). Given the nerve’s critical role, accurate targeting during local anesthetic delivery is essential to ensure complete unilateral maxillary analgesia (Castejón-González & Reiter, 2019).

In recent years, cone-beam computed tomography (CBCT) has emerged as a valuable tool in both human and veterinary dental practice (Stewart et al., 2021; Sasser, 2023). Introduced to U.S. dental markets in 2001, CBCT employs a cone-shaped X-ray beam and a two-dimensional detector to acquire volumetric data, generating high-resolution, isotropic voxel images across multiple planes (Abdelkarim, 2019). Compared to conventional CT, CBCT offers numerous advantages, including enhanced image detail, reduced radiation exposure, shorter scan times, and reduced artifacts attributes that have facilitated its rapid adoption in human dentistry (Heney et al., 2019). These benefits are increasingly being recognized in veterinary medicine, where CBCT enables precise anatomical visualization for diagnostic and interventional procedures (Duro et al., 2025). In veterinary medicine, CBCT has become valuable for detailed assessment of maxillofacial structures, including evaluation of dental pathology, identification of infraorbital and maxillary canal morphology, detection of root fractures, and preoperative planning for oral and craniofacial surgery (Van Thielen et al., 2012). Its high spatial resolution and reduced artifact formation allow clinicians to visualize bony landmarks with greater accuracy than conventional radiography, making it particularly suitable for studies involving regional anesthesia techniques where precise anatomical understanding is essential (Riggs et al., 2016; Ahmad et al., 2012).

Multiple maxillary nerve block approaches have been described in the veterinary literature (Bardell & Mosing, 2010), including the percutaneous subzygomatic approach (Pascoe & Chohan, 2020), infraorbital and deep infraorbital techniques (Viscasillas et al., 2013), intraorbital methods (Langton & Walker, 2017), and intraoral approaches at the level of the maxillary tuberosity (Thatcher et al., 2023). Deep infraorbital approaches, often delivered intraorally, involve advancing a needle through the infraorbital canal to the level of the first molar. Proper technique includes aspiration to avoid intravascular injection and slow deposition of the anesthetic to confine the agent within the canal (Niemiec et al., 2020). Modified techniques using venous catheters have also been reported but may increase the risk of complications (McCarthy & Chau, 2020).

Despite the widespread clinical use of these techniques, there remains a lack of comprehensive anatomical and radiographic studies comparing their efficacy, anesthetic spread, and associated risks (Foster et al., 2021). Key questions persist regarding the volume of anesthetic required, the consistency of diffusion along the nerve, and the resulting duration and completeness of analgesia. Given this uncertainty, practitioners often rely on personal experience rather than evidence-based criteria when selecting a technique (Beeston et al., 2022).

Therefore, the objective of the present study was to perform an anatomical and CBCT-based evaluation of three distinct approaches to maxillary nerve blockade in dogs: the percutaneous (subzygomatic), infraorbital, and intraoral (maxillary tuberosity) techniques. By assessing the extent of anesthetic diffusion and anatomical accuracy using methylene blue and contrast-enhanced imaging, this study aims to clarify the relative efficacy of each method, offering evidence-based guidance for clinical application in veterinary anesthesia.

## Material and methods

### Ethical statements

All protocols and procedures were in compliance with international guidelines for care and use of laboratory animals, and they were approved by Shahid Chamran University of Ahvaz (EE/99.3.02.69104/ scu.ac.ir). This randomized, blinded, cadaveric study was conducted using six canine heads obtained postmortem from adult mixed-breed dogs euthanized for reasons unrelated to the research. All experimental methods were performed in accordance with ARRIVE guidelines. All methods were carried out in accordance with relevant guidelines and regulations. Euthanasia was not conducted during the experimental period. The selection of six canine cadaver heads (*n* = 6) was guided primarily by ethical considerations and the principles of the 3Rs, Replacement, Reduction, and Refinement. Because all specimens were obtained postmortem from dogs euthanized for reasons unrelated to research, no animals were sacrificed specifically for this study. Consistent with the Reduction principle, the number of cadaver heads limited to the minimum required to reliably evaluate anatomical injectate spread and contrast distribution while avoiding unnecessary use of animal tissues.

### Specimen collection and study design

**Fig. 1:**
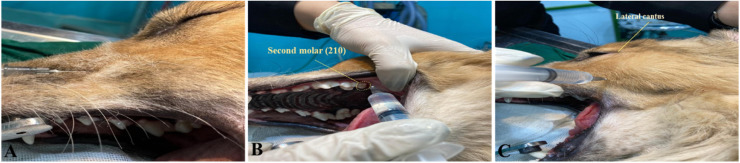
Maxillary nerve block techniques. (A) The infraorbital approach: Inserting the needle through the vestibular mucosa above the upper lip into the infraorbital foramen. (B) Maxillary tuberosity approach: The injection site is located behind the last molar (left 210 and right 110), with the molar highlighted using a yellow dotted line. (C) Subzygomatic approach: The injection site is demonstrated with the needle inserted perpendicularly below the lateral canthus and ventral to the rostral portion of the zygomatic arch.

All head specimens were separated within 1–5 days of euthanasia and stored at <4 °C until the time of experimentation. Exclusion criteria included brachycephalic breeds, age under six months, cranial trauma, or existing skull bone pathology. Each head was randomly assigned to receive one of three maxillary nerve block approaches: (1) infraorbital (IO), (2) maxillary tuberosity (MT), or (3) subzygomatic (SBZ). Each technique was performed on either the right or left side, while the contralateral side received one of the other approaches, ensuring bilateral injections in all specimens. Randomization of side allocation was performed before injection (Fig. 1).

### Standardization of injection techniques

Anatomical landmarks for the SBZ, MT, and IO approaches were identified using canine skull models before the experiment to standardize procedural consistency. Prior to performing the study injections, both veterinarians completed structured training that included practicing each technique on canine skull models and conducting pilot needle-placement sessions on non-study cadaver heads under supervision to ensure consistent mastery of landmarks, angles, and depths. To eliminate operator bias, injections were performed by two veterinarians without prior experience in the three techniques. The same solution was injected into the samples according to their groupings, that is, the infraorbital canal (Pascoe, 2016), the maxillary tuberosity (Gracis, 2013), and the subzygomatic approach (Gutiérrez Bautista et al., 2024). Per each side, 0.5 mL was injected from total volume of 3.2 mL of solution, composed of 0.5 mL bupivacaine 0.5 %, 1.1 mL distilled water, 1.6 mL iohexol (300 mg I/mL), and methylene blue 1 % per side. Injection speed was standardized by delivering each 0.5 mL aliquot over approximately 10 s, and negative-pressure aspiration was performed before each injection across all approaches to verify nonvascular placement. The injected volume of 0.5 mL per side was selected based on volumes commonly recommended in veterinary dental and anesthetic protocols for maxillary nerve blocks in dogs, particularly in cadaveric and controlled experimental settings. This conservative volume was chosen to standardize injections across all approaches and to allow comparison of technique-related diffusion patterns while minimizing excessive or nonselective spread.

### Infraorbital nerve block procedure

The infraorbital approach (Fig. 1-A), involved inserting the needle through the vestibular mucosa above the upper lip, into the infraorbital foramen, and advancing it through the infraorbital canal to the maxillary foramen, targeting the pterygopalatine fossa (Pascoe, 2016).

### Maxillary tuberosity approach procedure

For the maxillary tuberosity approach (Fig. 1-B), performed intraorally, a needle was inserted caudal to the last molar and directed perpendicular to the hard palate near the maxillary tuberosity, palpated manually. Mouth gags were used to maintain access. The depth of insertion ranged from 25 to 30 mm, depending on the skull size (Gracis, 2013).

### Subzygomatic nerve block technique

The subzygomatic technique (Fig. 1-C), required external palpation of the zygomatic arch below the globe. The needle was inserted perpendicularly to the skin and advanced to a depth sufficient to reach the pterygopalatine fossa. Aspiration preceded slow injection to minimize the risk of intravascular administration (Gutiérrez Bautista et al., 2024).

### CBCT imaging and analysis

CBCT scans were performed at Jundishapur University of Medical Sciences under the supervision of an oral and maxillofacial radiologist. Pre- and post-injection scans were taken bilaterally (Döring et al., 2018). Imaging was performed using a NewTom 5 G CBCT unit (QR Srl, Verona, Italy), operated at 110 kVp, 6.2 mA, and an exposure time of 3.6 s, parameters comparable to those commonly reported in veterinary maxillofacial imaging. A field of view (FOV) of 15 × 12 cm was selected to include the entire maxilla and infraorbital region. Images were reconstructed with an isotropic voxel size of 0.2 mm, providing high-resolution visualization of osseous structures and contrast distribution. Imaging data were analyzed independently by two blinded observers using standardized viewing conditions (fixed monitor, identical lighting, 50 cm viewing distance). CBCT image analysis was independently performed by two observers who were blinded to the injection technique and treatment assignment. Measurements of maxillary nerve staining and diffusion length were obtained separately by each observer using standardized measurement protocols. Inter-observer reliability for the primary outcome measures was assessed using the intraclass correlation coefficient (ICC), which demonstrated excellent agreement between observers. NNT Viewer software (version 3) was used for the primary image review. DICOM files were exported and further processed using OnDemand3D software version 1.0.10 (Cybermed Inc., Seoul, Cybermed Inc., Seoul, Korea) for reconstruction and measurement. The extent of contrast medium spread was assessed in both sagittal and dorsal planes at 1 mm and 10 mm slice thicknesses. Measurements included the linear spread of the contrast along the nerve pathways.

### Anatomical dissection and evaluation

Following CBCT analysis, each head underwent meticulous anatomical dissection performed by a third, blinded veterinarian. The skin and muscles over the temporomandibular joint, maxilla, and mandible were removed using scalpels and forceps. The mandible was excised by transecting the ramus below the condylar process. The zygomatic arch was dissected and transected at both the zygomatic and temporal process junctions. Soft tissues and connective structures around the pterygopalatine fossa and infraorbital canal were carefully removed to expose the maxillary nerve. Staining of the nerve was scored semi-quantitatively based on visual assessment: 0 = no staining (failure), 1 = partial staining (moderate), and 2 = full staining (ideal), as previously described by Becerra et al. (Becerra et al., 2018).

### Statistical analysis

Quantitative data were analyzed using GraphPad Prism 9.0.0. Descriptive statistics (means and standard deviations) were calculated for all continuous variables. Data normality was assessed using the Shapiro–Wilk test. For normally distributed variables, paired *t*-tests were used to compare dye spread between sides, and repeated-measures ANOVA with Bonferroni correction was applied for multiple comparisons among the three injection techniques. Friedman tests were used for non-parametric comparisons of staining intensity. Statistical significance was set at *p* < 0.05 for all tests.

## Results

### Distribution of injection approaches

**Fig. 2:**
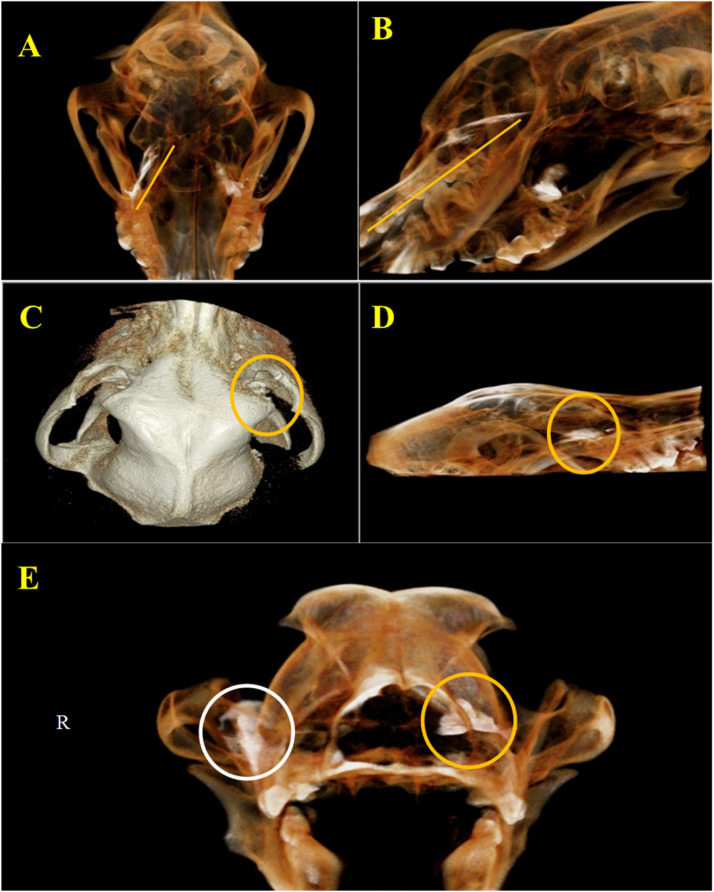
Contrast-enhanced CBCT images of an adult mixed-breed male dog's skull in 3D view. (A) Ventral view showing the progression of contrast media in the infraorbital (IO) approach to the maxillary nerve (yellow lines). (B) Lateral view illustrating the same distribution. (C) Dorsal view demonstrating the diffusion of contrast media at the pterygopalatine fossa using the maxillary tuberosity (MT) approach (yellow circles). (D)Lateral view of the MT approach diffusion pattern. (E) Rostral view depicting bilateral contrast media diffusion: the yellow circle indicates the MT approach on the left side, and the white circle highlights the spread associated with the subzygomatic approach on the right side.

Each of the three nerve block techniques including infraorbital (IO), maxillary tuberosity (MT), and subzygomatic (SBZ) was performed four times as per the randomized study design. All specimens were of uniform size and were analyzed using cone-beam computed tomography (CBCT) imaging in both sagittal and dorsal planes (Fig. 2). Measurements were taken at 1 mm and 10 mm slice thicknesses to assess contrast medium spread. However, the exact localization of the maxillary nerve could not be directly confirmed.

### Sagittal and dorsal plane analysis (1 mm thickness)

**Fig. 3:**
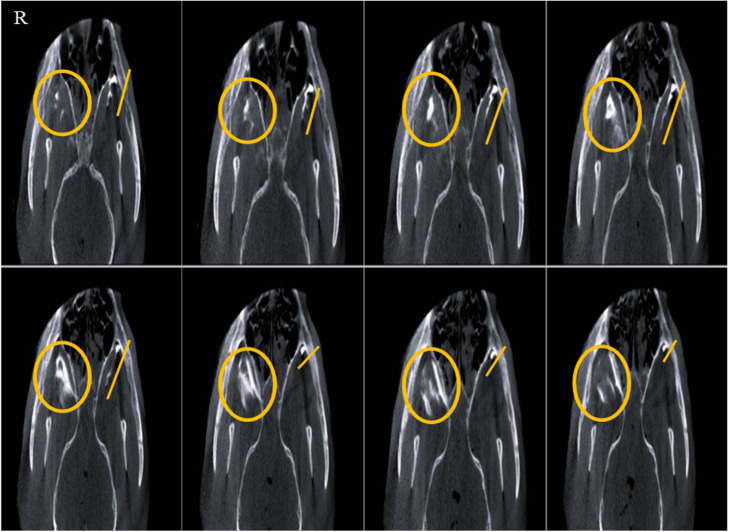
Contrast-enhanced CBCT of an adult mixed-breed male dog's skull, dorsal view. Yellow lines illustrate the progression of contrast media via the infraorbital (IO) approach on the left side, while yellow circles indicate the diffusion pattern of contrast media through the maxillary tuberosity (MT) approach on the right side.

Quantitative analysis revealed that the extent of contrast medium propagation along the maxillary nerve in the sagittal view at 1 mm thickness was significantly greater in the IO group compared to the MT and SBZ groups (*p* = 0.0036 and *p* = 0.0299, respectively) (Table 1). Similarly, in the dorsal view with 1 mm thickness, the IO approach demonstrated significantly greater contrast spread along the nerve than both the MT and SBZ groups (*p* = 0.0246 and *p* = 0.0168, respectively) (Fig. 3; Table 2).

**Table 1:** The maximum advance of the contrast Medium along the maxillary nerve length in sagittal view of 3 groups of CBCT images with a thickness of 1 and 10 mm.

Approaches	Sagittal view	
	1 mm thickness	10 mm thickness
**Infraorbital**	43.82 ± 2.745^⁎#^	21.04 ± 0.3401*
**Maxillary Tuberosity**	29.04 ± 3.233^#^	31.93 ± 0.7112
**Sub Zygomatic**	25.71 ± 3.221^⁎#^	23.16 ± 2.176*

*P < 0.05, #*P* < 0.05 in each row and column indicate significant differences, respectively.

**Table 2:** The maximum advance of the contrast Medium along the maxillary nerve length in dorsal view of 3 groups of CBCT images with a thickness of 1 and 10 mm.

Approaches	Dorsal view	
	1 mm thickness	10 mm thickness
**Infraorbital**	16.97 ± 0.6542*	46.65 ± 0.5629^⁎#^
**Maxillary Tuberosity**	23.07 ± 1.389*	37.97 ± 5.133^⁎#^
**Sub Zygomatic**	24.46 ± 2.697	21.08 ± 0.4043^#^

*P < 0.05, #*P* < 0.05 in each row and column indicate significant differences, respectively.

### Sagittal and dorsal plane analysis (10 mm thickness)

**Fig. 4:**
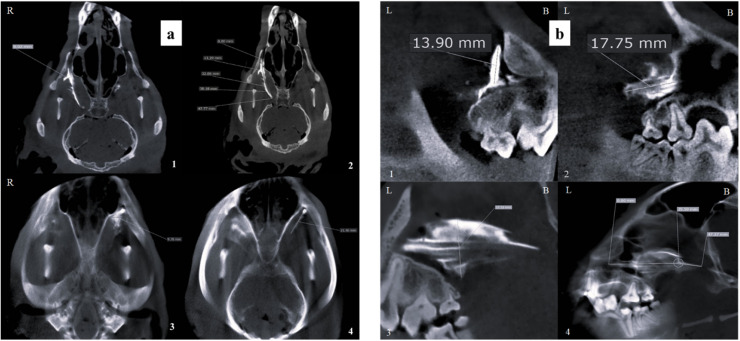
Contrast-enhanced CBCT images of an adult mixed-breed male dog's skull showing the distribution of contrast media in the infraorbital (IO) approach. (a) Dorsal view illustrating the width and length of contrast media diffusion at 1 mm and 10 mm thicknesses, respectively. (b) Sagittal view demonstrating similar diffusion dimensions with clear labeling of lateral (L) and buccal (B) orientations. Measurements indicate: 1. Width of contrast media diffusion at 1 mm thickness, 2. Length at 1 mm thickness, 3. Width at 10 mm thickness, and 4. Length at 10 mm thickness. *R* = right.

**Fig. 5:**
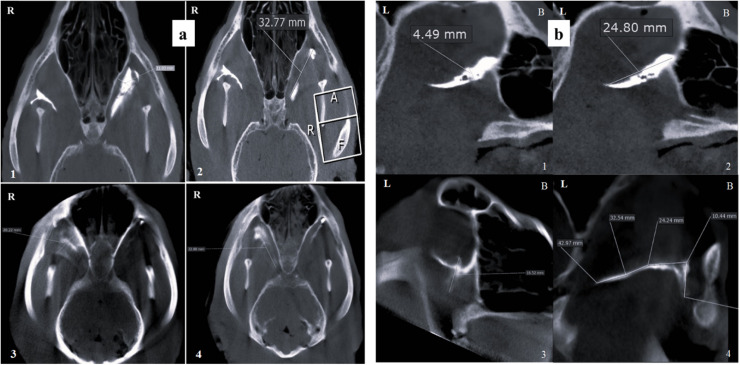
Contrast-enhanced CBCT images of an adult mixed-breed male dog's skull depicting the distribution of contrast media following the maxillary tuberosity (MT) approach. (a) Dorsal view showing: 1. Width of contrast media diffusion at 1 mm thickness, 2. Length at 1 mm thickness, 3. Width at 10 mm thickness, and 4. Length at 10 mm thickness. (b) Sagittal view presenting the same diffusion dimensions, with anatomical orientation indicators: *L* = lateral, *B* = buccal.

**Fig. 6:**
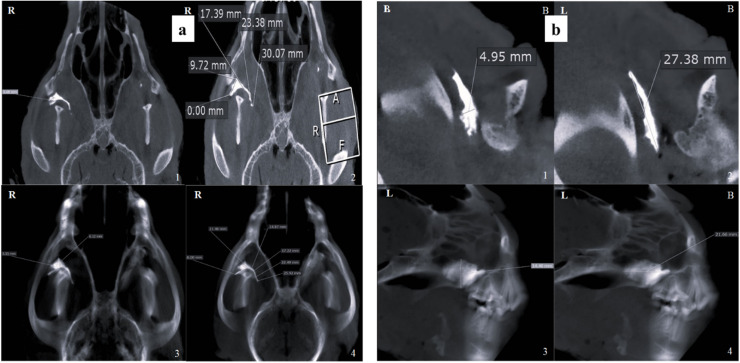
Contrast-enhanced CBCT images of an adult mixed-breed male dog's skull illustrating the diffusion of contrast media via the subzygomatic approach. (a) Dorsal view showing: 1. Width of contrast media diffusion at 1 mm thickness, 2. Length at 1 mm thickness, 3. Width at 10 mm thickness, and 4. Length at 10 mm thickness. (b) Sagittal views presenting the same measurements, with anatomical orientation markers: *L* = lateral, *B* = buccal.

When evaluated with a 10 mm slice thickness in the sagittal plane, contrast medium diffusion was significantly higher in the IO group compared to the MT group (*p* = 0.0001), and greater in the MT group than in the SBZ group (*p* = 0.0038). In the dorsal view at 10 mm thickness, a similar trend was observed. The IO group showed a significantly more extensive contrast spread compared to the SBZ group (*p* = 0.0001), while the MT group also demonstrated a significant advantage over the SBZ group (*p* = 0.0185) (Fig. 4, Fig. 5, Fig. 6; Tables 1, 2).

### Injection site width assessment

The width of contrast medium distribution at the injection site, observed in sagittal view at 1 mm thickness, was significantly greater in the MT group compared to the SBZ group (*p* = 0.0328) (Tables 3, 4).

**Table 3:** The maximum advance of the contrast Medium in the width of injection site in sagittal view of 3 groups of CBCT images with a thickness of 1 and 10 mm.

Approaches	Sagittal view	
	1 mm thickness	10 mm thickness
**Infraorbital**	10.87 ± 2.294	15.49 ± 1.385
**Maxillary Tuberosity**	3.675 ± 0.730*	14.50 ± 1.556*
**Sub Zygomatic**	8.745 ± 3.403*	15.56 ± 0.8082*

*P < 0.05 in each row indicates significant differences.

**Table 4:** The maximum advance of the contrast Medium in the width of injection site in dorsal view of 3 groups of CBCT images with a thickness of 1 and 10 mm.

Approaches	Dorsal view	
	1 mm thickness	10 mm thickness
**Infraorbital**	6.388 ± 2.397*	9.133 ± 0.4495*
**Maxillary Tuberosity**	12.15 ± 1.157	18.01 ± 2.321
**Sub Zygomatic**	6.193 ± 1.061	14.01 ± 6.736

*P < 0.05 in each row indicates significant differences.

### Comparative nerve staining between approaches

**Fig. 7:**
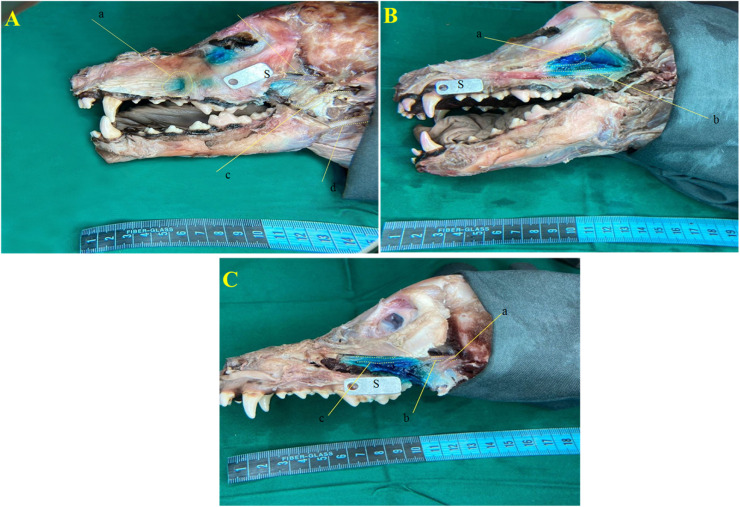
Methylene blue dye progression following three different approaches in an adult mixed-breed male dog's skull. (A) Infraorbital (IO) approach showing anatomical landmarks: a. infraorbital foramen, b. maxillary nerve, c. mandibular nerve, d. facial nerve. (B) Maxillary tuberosity (MT) approach highlighting: a. zygomatic gland, b. maxillary nerve. (C) subzygomatic approach displaying: a. ophthalmic nerve, b. maxillary nerve, c. infraorbital nerve. *S* = scale.

Staining of the maxillary nerve was significantly more pronounced in the IO group compared to both the MT (*p* = 0.0002) and SBZ (*p* = 0.0004) groups. Additionally, a significant difference in staining was identified between the MT and SBZ groups (*p* = 0.0011). However, no statistically significant differences were observed in staining intensity among the three approaches (*p* < 0.05) (Fig. 7; Table 5).

**Table 5:** Comparison of the progress of methylene blue dye and the intensity of staining of maxillary nerve in three groups studied.

Approaches	Progress of staining along maxillary nerve	staining intensity
**Infraorbital**	63.2 ± 1.6*	1.8 ± 0.3
**Maxillary Tuberosity**	46.0 ± 1.1*	1.5 ± 0.4
**Sub Zygomatic**	23.8 ± 1.8*	1.4 ± 0.5

*P < 0.05 in each column indicates significant differences.

### Correlation between radiological and anatomical data

A comparative analysis of radiological measurements (Table 1, Table 2) with anatomical observations revealed that the IO approach exhibited the closest correlation with anatomical dye diffusion lengths in dorsal views at 10 mm thickness and sagittal views at 1 mm thickness. In contrast, the MT approach aligned most closely with anatomical diffusion lengths in both dorsal and sagittal views at 10 mm thickness. For the SBZ approach, the best anatomical correlation was observed in sagittal views at a 10 mm thickness and in dorsal views at a 1 mm thickness.

## Discussion

This study appears to be the first to quantitatively assess contrast medium distribution via cone-beam computed tomography (CBCT) following maxillary nerve block injections in canine cadavers. No previous studies have employed CBCT to evaluate contrast dispersion along the maxillary nerve following intraoral (IO), maxillary tuberosity (MT), or subzygomatic (SBZ) approaches in dogs. The current findings revealed that contrast medium progression along the maxillary nerve, particularly in the sagittal plane at 1 mm slice thickness, was significantly greater in the IO group compared to both MT and SBZ groups. A similar pattern was observed in the dorsal view, again highlighting the superior distribution achieved via the IO approach. Radiographically, the IO approach demonstrated a linear pattern of contrast distribution, consistent with the injection site being within the infraorbital canal. This contrasts with the MT and SBZ approaches, where the distribution patterns suggested dispersion within the surrounding soft tissues rather than within a neurovascular conduit. The disparity in distribution patterns likely reflects the anatomical differences in needle placement, with IO injections more reliably entering the infraorbital canal and the others depositing contrast medium adjacent to the target nerve. The anatomical measurements and radiographic interpretations suggest that the observed discrepancies in distribution length may also result from radiographic superimposition of bony structures, particularly in the IO group. This overlap complicates direct comparisons of measured diffusion lengths across approaches, though the linearity and extent of contrast spread in the IO group clearly indicate more efficient nerve staining. To provide additional context for our primary comparisons, post-hoc power analyses were performed using GPower 3.1 (Heinrich-Heine-Universität Düsseldorf, Germany) for the main outcomes comparing infraorbital (IO) versus maxillary tuberosity (MT), IO versus subzygomatic (SBZ), and MT versus SBZ approaches. The analyses indicated moderate to high observed power (0.72–0.89) despite the small sample size (*n* = 6 cadavers), reflecting the large effect sizes observed for nerve staining and diffusion length. While the limited number of cadavers remains a key limitation, these results suggest that the detected differences are likely robust and provide meaningful insights for clinical technique selection.

Recent human clinical research has consistently demonstrated that CBCT provides superior visualization of neural canals, accessory foramina, and fine maxillofacial structures compared with conventional radiography (Yasin et al., 2025). Studies evaluating maxillary and mandibular canal anatomy have shown that CBCT enables highly accurate detection of canal trajectories, foraminal variations, and neurovascular pathway morphology, thereby supporting its use for pre-procedural planning and risk minimization (Uthman et al., 2024; Shetty et al., 2021). In alignment with our findings, Viscasillas et al. reported successful staining of the maxillary nerve using CT imaging after dorsal extraconal block injections via a temporal approach, highlighting the importance of precise needle placement near the orbital fissure. Their study also observed nerve staining when contrast reached the orbital fissure, suggesting partial blockade of the maxillary nerve (Viscasillas et al., 2019). Beeston et al. used CT imaging to assess the IO block and recommended perpendicular needle insertion to the palate, posterior to the second maxillary molar, and aligned with its midline. They emphasized the significance of needle penetration depth, recommending measurements based on individual jaw width for optimal placement practices relevant to our IO approach findings (Beeston et al., 2022). Similarly, Davis et al. compared IO and SBZ methods using CT and found contrast medium only within the infraorbital canal for the IO approach, echoing our CBCT observations (Davis et al., 2021). In equine models, Weber et al. confirmed diffusion of contrast along the infraorbital canal to the maxillary foramen in 90 % of cases, again using CT (Weber et al., 2020). In contrast, our CBCT analysis demonstrated 100 % contrast detection, suggesting a diagnostic advantage of CBCT in evaluating perineural diffusion. CBCT's enhanced sensitivity was further corroborated by repeated confirmations of contrast medium at the injection site, surpassing the reliability shown in prior CT studies (Weber et al., 2020). These results support CBCT as a superior modality for visualizing the anatomic and diffusion characteristics of injected agents in veterinary craniofacial applications. A potential limitation of the imaging component of this study relates to partial-volume effects associated with the use of 1 mm versus 10 mm slice thicknesses. Thicker slices may artificially increase the apparent continuity of contrast diffusion, whereas thinner slices may underrepresent spread due to reduced voxel averaging. These factors should be considered when interpreting CBCT-based diffusion measurements, although the overall comparative patterns between techniques remained consistent across slice thicknesses.

The use of ultrasound in maxillary nerve blocks has also been extensively documented. Hagag et al. demonstrated successful dye diffusion without vascular compromise using ultrasound guidance in both cadavers and live donkeys (Hagag et al., 2018). In contrast, anatomical landmarks alone, as employed in our study, yielded more variable results. Stauffer et al. and O’Neill et al. also confirmed improved outcomes using ultrasonographic guidance, even among inexperienced operators (O’Neill et al., 2014; Stauffer et al., 2017). Despite the absence of ultrasound guidance in this study, injections were guided solely by anatomical landmarks. The combination of bupivacaine, iohexol contrast medium, and methylene blue dye allowed comprehensive visualization of injection outcomes. Measurements of maxillary nerve length and contrast diffusion across sagittal and dorsal views (at 1 mm and 10 mm thicknesses) provided reliable, realistic assessments of each technique’s effectiveness. Among the three techniques evaluated, the IO approach consistently demonstrated superior results in terms of both staining length and dye uptake. The MT and SBZ approaches followed in effectiveness, with MT achieving greater staining than SBZ. These findings align with the underlying anatomical assumptions regarding each approach’s proximity and access to the infraorbital canal. Viscasillas et al. previously described a modified IO technique using a venous catheter and intraoral advancement under CT guidance, which resulted in greater maxillary nerve staining than cutaneous approaches (Viscasillas et al., 2013). This method’s success reinforces our findings, particularly in terms of staining length and contrast distribution. Although a concurrent in vivo clinical evaluation to assess analgesic efficacy was not feasible in this study due to hardware limitations, our anatomical and radiographic outcomes provide strong indirect evidence of block effectiveness.

Fizzano et al. also demonstrated effective nerve block via a modified IO approach during rhinoscopy and nasal biopsy in dogs, with 3 mL of methylene blue sufficient to stain all major branches of the maxillary nerve within the pterygopalatine fossa (Fizzano et al., 2017). The reduced contrast dispersion observed in our study may be attributed to the lower volume of injectate used and the absence of modified techniques. The findings by Henry et al. supported the superior accuracy of intraoral methods compared to extraoral techniques (Henry et al., 2014). Likewise, Langton and Walker validated the efficacy of the transorbital approach in canine models, though no significant advantage over SBZ was detected (Langton & Walker, 2017). Davis et al. noted no statistical difference in stained nerve lengths between IO and SBZ in feline models, although the IO approach required less injectate volume for effective staining (Davis et al., 2021). Concerns about ocular trauma following dental anesthesia have been raised by Volk et al. who advised against needle advancement into the infraorbital canal during maxillary tooth extractions (Volk et al., 2019). The findings of Shilo-Benjamini et al. and Chohan & Pascoe further confirmed that the infraorbital canal approach results in more prolonged and effective maxillary nerve anesthesia compared to alternative approaches (Shilo-Benjamini et al., 2022; Chohan & Pascoe, 2021). In our study, although the infraorbital approach produced the greatest and most extensive contrast diffusion, it is important to recognize the potential clinical risks associated with infraorbital needle placement. Excessive advancement within or toward the orbital cavity may increase the risk of retrobulbar trauma or inadvertent ocular penetration, particularly in smaller dogs or breeds with shallow infraorbital canals. To minimize these risks in practice, clinicians should pre-measure expected insertion depth, maintain a shallow needle angle, use appropriately sized needles, and perform gentle aspiration before injection. When anatomical landmarks are difficult to identify, adjunctive imaging such as ultrasound may provide additional safety and precision. In our study, although the infraorbital approach produced the greatest and most extensive contrast diffusion, it is important to recognize the potential clinical risks associated with infraorbital needle placement. Excessive advancement within or toward the orbital cavity may increase the risk of retrobulbar trauma or inadvertent ocular penetration, particularly in smaller dogs or breeds with shallow infraorbital canals. To minimize these risks in practice, clinicians should pre-measure expected insertion depth, maintain a shallow needle angle, use appropriately sized needles, and perform gentle aspiration before injection. When anatomical landmarks are difficult to identify, adjunctive imaging such as ultrasound may provide additional safety and precision.

A critical observation from this study lies in the differential spread and efficacy of contrast medium among the IO, MT, and SBZ techniques. The intraoral (IO) approach consistently yielded superior staining, both in terms of distribution pattern and staining length, across all radiographic views. These findings align with the anatomical premise that intraoral access offers more direct proximity to the infraorbital canal, thereby facilitating more effective perineural delivery (Li et al., 2020). The success of IO method appears to hinge on both the route’s anatomical alignment and the reduced resistance provided by mucosal tissue, as opposed to the dermal and muscular structures encountered in extraoral routes like SBZ. In contrast, the maxillary tuberosity (MT) approach, while showing moderate efficacy, displayed less predictable staining, likely due to variability in injection depth and anatomical variation among cadavers. This method may also introduce a risk of inadvertent vascular puncture, given the regional vascular complexity. The SBZ method, although commonly employed due to its external accessibility, resulted in the least consistent nerve staining and showed more diffused contrast patterns, suggesting potential extraneural deposition. These results strongly support the hypothesis that anatomical guidance alone may not be sufficient for precise needle placement in more externally approached techniques, such as MT and SBZ, thereby advocating for the integration of adjunctive imaging modalities in clinical settings to enhance precision and consistency. While the present study was performed on cadaveric specimens, the anatomical and radiological markers assessed particularly the extent and pattern of contrast spread along the maxillary nerve serve as meaningful proxies for predicting clinical efficacy. In regional anesthesia, successful perineural deposition is strongly associated with improved onset, duration, and reliability of sensory blockade in live patients (Desai et al., 2021). The superior and more directed diffusion observed with the infraorbital (IO) approach suggests that, in clinical practice, this technique is likely to produce a more consistent and complete maxillary nerve block compared to the MT and SBZ approaches. Although functional endpoints such as analgesic duration or behavioral responses cannot be evaluated in a cadaveric model, the correlation between injectate spread and nerve staining provides a well-established foundation for anticipating clinical performance. This study was conducted using a cadaveric model, which inherently limits its ability to fully replicate clinical conditions. Specifically, this model does not allow assessment of nerve block onset time, duration of analgesia, or overall clinical efficacy in live animals, nor does it permit evaluation of potential complications that could arise during actual veterinary procedures. Additionally, while radiological contrast diffusion provides valuable information regarding perineural deposition of injectate, it is an indirect proxy and cannot be assumed to guarantee effective anesthesia in clinical settings. Therefore, although our findings offer important comparative anatomical insights, further studies in live animals are required to confirm analgesic efficacy and procedural safety.

## Conclusion

This study demonstrated that the IO approach offers superior efficacy compared to the MT and SBZ approaches for maxillary nerve blocks in dogs. CBCT analysis revealed that the IO technique provided the greatest contrast medium diffusion length with the least diffusion width, indicating more targeted and effective anesthetic delivery. Furthermore, the IO and MT approaches allowed for accurate measurement of diffusion length using dorsal view slices with 10 mm thickness, while the SBZ approach permitted similar assessment in sagittal view. These findings support the IO approach as the most precise and effective technique among those evaluated. Future studies should investigate the clinical efficacy and safety of these approaches in live subjects to further validate the anatomical and radiological findings.

## Funding

This work was supported by Shahid Chamran University of Ahvaz, Iran [Grant number: SCU, VB1401.293].

## Data availability

The data supporting this study’s findings are available from the corresponding author upon reasonable request.

## Ethical

All protocols and procedures were in compliance with international guidelines for care and use of laboratory animals, and they were approved by Shahid Chamran University of Ahvaz (EE/99.3.02.69104/ scu.ac.ir).

## CRediT authorship contribution statement

**Kaveh Khazaeel:** Writing – review & editing, Supervision, Resources, Project administration, Data curation, Conceptualization. **Anahita Memardezfouli:** Writing – original draft, Software, Methodology, Investigation. **Hadi Imani Rastabi:** Writing – review & editing, Writing – original draft, Supervision, Software, Resources, Methodology, Investigation. **Seyed Arman Mohagheghi:** Writing – review & editing, Writing – original draft, Software, Resources, Methodology.

## Declaration of competing interest

The authors declare that they have no known competing financial interests or personal relationships that could have appeared to influence the work reported in this paper.
